# Comparison of the 2015 and 2023 Duke–European Society of Cardiology Criteria Among Patients With Suspected Infective Endocarditis

**DOI:** 10.1093/cid/ciae370

**Published:** 2024-07-12

**Authors:** Matthaios Papadimitriou-Olivgeris, Pierre Monney, Michelle Frank, Georgios Tzimas, Nicolas Fourre, Virgile Zimmermann, Piergiorgio Tozzi, Matthias Kirsch, Mathias Van Hemelrijck, Jana Epprecht, Benoit Guery, Barbara Hasse

**Affiliations:** Infectious Diseases Service, Lausanne University Hospital and University of Lausanne, Lausanne, Switzerland; Infectious Diseases Service, Cantonal Hospital of Sion and Institut Central des Hôpitaux, Sion, Switzerland; Department of Cardiology, Lausanne University Hospital and University of Lausanne, Lausanne, Switzerland; Department of Cardiology, University Hospital Zurich and University of Zurich, Zurich, Switzerland; Department of Cardiology, Lausanne University Hospital and University of Lausanne, Lausanne, Switzerland; Infectious Diseases Service, Lausanne University Hospital and University of Lausanne, Lausanne, Switzerland; Infectious Diseases Service, Lausanne University Hospital and University of Lausanne, Lausanne, Switzerland; Department of Cardiac Surgery, Lausanne University Hospital and University of Lausanne, Lausanne, Switzerland; Department of Cardiac Surgery, Lausanne University Hospital and University of Lausanne, Lausanne, Switzerland; Department of Cardiac Surgery, University Hospital Zurich and University of Zurich, Zurich, Switzerland; Department of Infectious Diseases and Hospital Epidemiology, University Hospital Zurich and University of Zurich, Zurich, Switzerland; Infectious Diseases Service, Lausanne University Hospital and University of Lausanne, Lausanne, Switzerland; Department of Infectious Diseases and Hospital Epidemiology, University Hospital Zurich and University of Zurich, Zurich, Switzerland

**Keywords:** infective endocarditis, Duke criteria, European Society of Cardiology, valve leaflet thickening, spondylodiscitis

## Abstract

**Background:**

Diagnosing infective endocarditis (IE) poses a significant challenge. This study aimed to compare the diagnostic accuracy of the 2015 and 2023 Duke clinical criteria introduced by the European Society of Cardiology (ESC) in a cohort of patients suspected of having IE.

**Methods:**

Conducted retrospectively at 2 Swiss university hospitals between 2014 and 2023, the study involved patients with suspected IE. Each hospital’s endocarditis team categorized cases as either IE or not IE. The performance of each iteration of the Duke-ESC clinical criteria was assessed based on the agreement between definite IE and the diagnoses made by the endocarditis team.

**Results:**

Among the 3127 episodes of suspected IE, 1177 (38%) were confirmed to have IE. Using the 2015 Duke-ESC criteria, 707 (23%) episodes were deemed definite IE, with 696 (98%) receiving a final IE diagnosis. With the 2023 Duke-ESC criteria, 855 (27%) episodes were classified as definite IE, of which 813 (95%) were confirmed as IE. The 2015 and 2023 Duke-ESC criteria categorized 1039 (33%) and 1034 (33%) episodes, respectively, as possible IE. Sensitivity for the 2015 Duke-ESC and the 2023 Duke-ESC criteria was calculated at 59% (95% confidence interval, 56%–62%), and 69% (66%–72%), respectively, with specificity at 99% (99%–100%), and 98% (97%–98%), respectively.

**Conclusions:**

The 2023 Duke-ESC criteria demonstrated significant improvements in sensitivity compared to the 2015 version, although one-third of episodes were classified as possible IE by both versions.

## Teaser

Among 3127 episodes of suspected infective endocarditis (IE), the 2023 Duke–European Society of Cardiology (ESC) clinical criteria showed higher sensitivity (69%) compared to 2015 Duke-ESC (59%) criteria for IE diagnosis; their specificity was comparable (98% and 99%, respectively).

Establishing a consensus on the definition of infective endocarditis (IE) is crucial for maintaining research quality. Therefore, the Duke criteria were developed with the aim of standardizing the diagnosis of IE [1, 2]. The criteria have undergone several revisions over the years. In 2015, the European Society of Cardiology (ESC) updated the criteria, integrating fluorine 18–labeled fluorodeoxyglucose positron emission tomography/computed tomography (^18^F-FDG PET/CT) for diagnosing prosthetic valve IE [2, 3]. Subsequently, in 2023, the International Society for Cardiovascular Infectious Diseases (ISCVID) made further modifications to the criteria. These changes included adjustments to the microbiology criteria, broadening the application of ^18^F-FDG PET/CT in native valve and cardiac implantable electronic device (CIED) lead IE, and introducing surgical evidence of IE as a new major criterion [4]. Several validation studies found that the 2023 Duke-ISCVID criteria yielded higher sensitivities compared than 2015 Duke-ESC criteria (80%–98% vs 70%–95%, respectively), but lower specificities (46%–98% vs 74%–100%) [5–10].

In 2023, the ESC again introduced a modified version of the Duke criteria [11]. Among the 5 new modifications, 3 mirrored those of the 2023 Duke-ISCVID criteria [4], encompassing changes to the cardiac predisposing criterion, microbiology, and imaging. The 2023 Duke-ESC criteria featured 2 modifications not present in the 2023 Duke-ISCVID criteria, which involved identifying valve thickening as a distinctive feature of IE and incorporating hematogenous osteoarticular complications into the vascular phenomena criterion [4, 11]. To date. only few studies evaluated the 2023 Duke-ESC criteria in small patient cohorts [5, 9, 12]. This study aims to evaluate the diagnostic accuracy of both the 2015 and 2023 Duke-ESC clinical criteria in a substantial Swiss patient cohort with bacteremia caused by any pathogen and displaying suspected cases of IE.

## METHODS

Researchers from Lausanne University Hospital (CHUV) and University Hospital Zurich (USZ) in Switzerland collaborated on a multicenter investigation. They combined data from 3 cohorts: cohort A, a cohort of patients from CHUV with suspected IE, spanning from January 2014 to June 2023. It involves retrospective inclusion of patients with IE from January 2014 to December 2017 and prospective inclusion of all patients with suspected IE from January 2018 to June 2023; cohort B, a cohort of patients from CHUV with bacteremia/candidemia, with retrospective inclusion of patients from January 2015 to December 2021; and cohort C, a cohort of patients from USZ with possible/proven IE, spanning from January 2014 to December 2022, with retrospective inclusion from January 2014 to December 2017 and prospective inclusion from January 2018 to December 2022.

Ethical approval was obtained from Swiss ethics committees (CER-VD 2017-02137 and CER-VD 2021-02516; KEK-2014-0461; BASEC 2017-01140). For the prospective cohort, written informed consent was obtained. For the retrospective inclusion of patients, the ethics committees waived the need of informed consent to participate. Eligible participants included adult patients aged ≥18 years with suspected IE from cohorts A–C. We defined patients with suspected IE as those who underwent blood cultures and echocardiography specifically to diagnose IE. Additional inclusion criteria involved the absence of data refusal for the retrospective cohort and written consent for the prospective one. We collected demographic, clinical, imaging, and microbiological information from patients’ health records. Each center established its endocarditis team in January 2018. From this date onward, the final diagnosis of IE at day 60 was determined based on microbiological, imaging, surgical, and pathological findings. Before 2018, each case underwent classification as IE or not, based on the assessment of 4 expert clinicians (M. P. O. and P. M. at CHUV; M. V. H. and B. H. at USZ). Cases were categorized as rejected, possible, or definite IE, following the 2015 Duke-ESC [2] and 2023 Duke-ESC [11] clinical criteria.

We used SPSS version 26.0 (SPSS) for the statistical analysis. We used the Mann-Whitney *U* test to assess continuous variables, and χ^2^ or Fisher exact tests used for categorical variables. Evaluating the performance of the Duke-ESC clinical criteria included comparing diagnoses provided by both the endocarditis teams or the expert clinicians (considered the reference standard), with cases identified as definite IE based on the criteria. We calculated sensitivity, specificity, positive and negative predictive values, and accuracy, with the corresponding 95% confidence intervals in the whole cohort. In line with the 2023 Duke-ISCVID proposition [4], sensitivity was also assessed in pathologically confirmed IE, in IE cases with positive valve cultures/molecular assay results matching the pathogen identified in blood cultures, and if positive CIED lead cultures matched the pathogen found in blood cultures [10]. All tests were 2 tailed, and differences were considered statistically significant at *P* < .05.

## RESULTS

**Figure 1.**
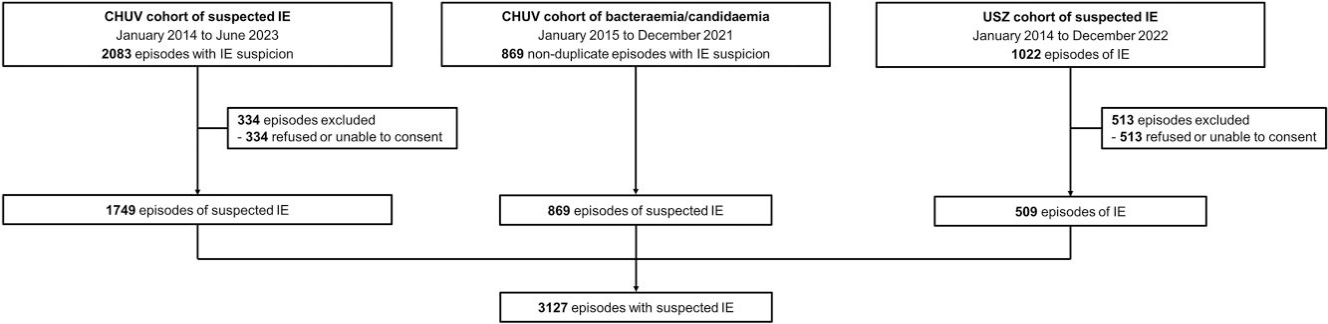
Flowchart of included patients from the 3 cohorts. Abbreviations: CHUV, Lausanne University Hospital; IE, infective endocarditis; USZ, University Hospital Zurich.

We analyzed 3127 episodes, consisting of 1749 suspected IE cases from the CHUV cohort, 869 nonduplicate bacteremia/candidemia episodes from the same hospital, and 509 from the USZ IE cohort (Figure 1). Of these cases, 1177 (38%) ultimately received a final IE diagnosis by each institution's endocarditis team or by the expert clinicians, respectively. Of the patients, 626 (36%) originated from cohort A, 42 (5%) from cohort B, and 509 (100%) from cohort C. Of the 1177 IE episodes, 752 (64%) were linked to native valves, 317 (27%) to prosthetic valves, 180 (15%) to CIED leads, and 7 (0.6%) to other intracardiac structures. Transthoracic echocardiography (TTE), transesophageal echocardiography (TEE), ^18^F-FDG PET/CT, and cardiac CT were performed in 2822 (90%), 1494 (48%), 468 (15%), and 84 (3%) episodes, respectively.

Table 1 presents patient characteristics stratified by the 2 iterations of the Duke-ESC criteria. Using the 2015 Duke-ESC clinical criteria, 707 episodes (23%) were classified as definite IE, with 696 (98%) receiving a final IE diagnosis. With the 2023 Duke-ESC clinical criteria, 855 episodes (27%) were categorized as definite IE, of which 813 (95%) received a final IE diagnosis. The 2015 criteria classified 1039 episodes (33%) as possible IE, while the 2023 criteria classified 1034 episodes (33%) similarly. Valve surgery, CIED extraction, and autopsy were performed in 479 episodes (15%), 137 of 528 (26%), and 27 (0.9%), respectively. Using both versions of the Duke-ESC criteria, the Duke pathological criterion was evident in episodes 311 (10%), whereby histopathology rejected the IE diagnosis in 27 episodes (0.9%).

**Table 1.** Patient Characteristics by Infective Endocarditis (IE) Diagnosis in 3127 Episodes of Suspected IE

Patient Characteristic	Episodes, No. (%)^a^		*P* Value
	No IE (n = 1950)	IE (n = 1177)	
Demographic			
Male sex	1329 (68)	879 (75)	<.001
Age, median (IQR), y	68 (55–78)	66 (51–75)	<.001
Cardiac predisposing factors			
Intravenous drug use	105 (5)	117 (10)	<.001
Rheumatic heart disease/hypertrophic cardiomyopathy	3 (0.2)	16 (1)	<.001
Congenital disease	35 (2)	215 (18)	<.001
Prosthetic valve	123 (6)	317 (27)	<.001
Prior endocarditis	51 (3)	147 (13)	<.001
CIED^b^	143 (7)	372 (32)	<.001
Transcatheter aortic valve replacement^b^	22 (1)	48 (4)	<.001
Heart transplantation^b^	21 (1)	4 (0.3)	.02
Left ventricular assist device^b^	15 (0.8)	6 (0.5)	.50
Minor predisposition criterion (Duke-ESC 2015)	269 (14)	592 (50)	<.001
Minor predisposition criterion (Duke-ESC 2023)	410 (21)	842 (72)	<.001
Microbiological data			
Bacteremia/fungemia	1540 (79)	1060 (90)	<.001
*Staphylococcus aureus*	692 (36)	458 (39)	.056
Coagulase-negative staphylococci	123 (6)	85 (7)	.34
*Streptococcus* spp.	340 (17)	320 (27)	<.001
*Streptococcus gallolyticus*	22 (1)	55 (5)	<.001
Viridans streptococci	200 (10)	192 (16)	<.001
*Enterococcus* spp.	120 (6)	133 (11)	<.001
Community-acquired enterococci without known primary focus^b^	21 (1)	118 (10)	<.001
* Enterococcus faecalis*^b^	71 (4)	84 (7)	<.001
Gram positive (other than staphylococci, streptococci, and enterococci)	49 (3)	43 (4)	.08
HACEK	2 (0.1)	29 (3)	<.001
Gram negative (other than HACEK)	286 (15)	33 (2)	<.001
Fungi	119 (6)	14 (1)	<.001
*Coxiella burnetii* antiphase I IgG titer ≥1:800	7 (0.4)	9 (0.8)	.13
Major microbiological criterion (Duke-ESC 2015)	648 (33)	773 (66)	<.001
Major microbiological criterion (Duke-ESC 2023)	675 (35)	763 (65)	<.001
Minor microbiological criterion (Duke-ESC 2015)	333 (17)	63 (5)	<.001
Minor microbiological criterion (Duke-ESC 2023)	283 (15)	165 (14)	.71
Imaging data			
Echocardiographic (TTE or TEE) findings positive for vegetation, perforation, abscess, aneurysm, pseudoaneurysm, or fistula	23 (1)	840 (71)	<.001
Abnormal metabolic activity seen with ^18^F-FDG PET/CT			
In prosthetic valve	3 (0.2)	76 (7)	<.001
In native or CIED lead^b^	2 (0.1)	45 (4)	<.001
Leaflet thickening seen with echocardiography (TTE or TEE)^b^	43 (2)	104 (9)	<.001
Cardiac CT findings positive for vegetation, perforation, abscess, aneurysm, pseudoaneurysm, or fistula	3 (0.2)	41 (4)	<.001
Leaflet thickening seen with cardiac CT^b^	4 (0.2)	9 (0.8)	.02
Major imaging criterion (Duke-ESC 2015)	25 (1)	885 (75)	<.001
Major imaging criterion (Duke-ESC 2023)	67 (3)	947 (81)	<.001
Manifestations			
Minor fever criterion (both Duke-ESC versions)	1548 (79)	920 (78)	.42
Vascular phenomena (major arterial emboli, septic pulmonary infarcts, mycotic aneurysm, intracranial hemorrhage, conjunctival hemorrhage, and Janeway lesions)	210 (11)	622 (53)	<.001
Hematogenous osteoarticular septic complications (spondylodiscitis, septic arthritis)^b^	181 (9)	137 (12)	.04
Spondylodiscitis^b^	89 (5)	68 (6)	.15
Septic arthritis^b^	105 (5)	80 (7)	.12
Minor vascular criterion (Duke-ESC 2015)	210 (11)	622 (53)	<.001
Minor vascular criterion (Duke-ESC 2023)	377 (19)	690 (59)	<.001
Minor immunological criterion (both Duke-ESC versions)	27 (1)	149 (13)	<.001
Data on surgery/CIED extraction/histopathology			
Valve surgery performed	18 (0.9)	461 (39)	<.001
CIED extraction (among 528 patients with CIED)	12 (8)	125 (33)	<.001
Autopsy performed	23 (1)	20 (2)	.27
Histopathology compatible for IE or positive culture of vegetation, abscess or embolized lesion	0 (0)	311 (26)	<.001
Duke pathological criterion (both versions)	0 (0)	311 (26)	<.001
Exclusion pathological criterion (both versions)	27 (1)	0 (0)	<.001
Classification as IE			
By Duke-ESC 2015 clinical criteria			
Rejected	1288 (66)	93 (8)	…
Possible	651 (33)	388 (33)	…
Definite	11 (0.6)	696 (59)	<.001
By Duke-ESC 2023 clinical criteria			
Rejected	1190 (61)	48 (4)	…
Possible	718 (37)	316 (27)	…
Definite	42 (2)	813 (69)	<.001

Abbreviations: ^18^F-FDG PET/CT, fluorine 18–labeled fluorodeoxyglucose positron emission tomography/computed tomography; CIED, cardiac implantable electronic device; ESC, European Society of Cardiology; HACEK, *Haemophilus* spp., *Aggregatibacter* spp., *Cardiobacterium hominis*, *Eikenella corrodens*, *Kingella kingae*; IE, infective endocarditis; IgG, immunoglobulin G; IQR, interquartile range; TEE, transesophageal echocardiography; TTE, transthoracic echocardiography.

^a^Data represent no. (%) of episodes unless otherwise specified.

^b^Characteristics that differ between the 2015 and 2023 Duke-ESC criteria.

We detected leaflet thickening in 158 episodes (5%), with a higher prevalence among episodes with IE than among those without (9% vs 2%, respectively; *P* < .001). In 75 episodes (2%), leaflet thickening was the sole typical lesion of IE. Of note, the occurrence of leaflet thickening alone was similar between episodes with and those without IE (42 of 1950 [2%] vs 33 of 1177 [3%], respectively; *P* = .28). We identified spondylodiscitis in 157 cases (5%) and septic arthritis in 185 (6%). Table 1 indicates that neither of these conditions was associated with the final diagnosis of IE. Among the 1117 cases with a final IE diagnosis, we found spondylodiscitis with similar frequency in patients or and without embolic events (34 of 622 [5%] vs 34 of 555 [6%], respectively; *P* = .71). The same was true for septic arthritis (41 of 622 [7%] vs 39 of 555 (7%); *P* = .82).

Table 2 illustrates the diagnostic performance of both versions of the Duke-ESC clinical criteria. The sensitivities for the 2015 Duke-ESC and the 2023 Duke-ESC criteria were calculated at 59% (95% confidence interval, 56%–62%), and 69% (66%–72%), respectively, with specificities of 99% (99%–100%), and 98% (97%–98%), respectively. Based on the ISCVID proposal, the sensitivities of both versions of the criteria were calculated in patients with confirmed IE. The 2023 Duke-ESC criteria for IE showed higher sensitivity (75%) compared than the 2015 version (67%) (Table 3). The increase in sensitivity was more pronounced in native valve IE episodes (81% for 2023 Duke-ESC vs 72% for 2015 Duke-ESC) than in prosthetic valve IE (73% vs 70%, respectively).

**Table 2.** Performance of 2015 and 2023 Duke–European Society of Cardiology Clinical Criteria in 3127 Episodes of Suspected Infective Endocarditis

Duke-ESC Criteria	Value (95% CI), %				
	Sensitivity	Specificity	PPV	NPV	Accuracy
2015	59 (56–62)	99 (99–100)	98 (97–99)	80 (79–81)	84 (83–85)
2023	69 (66–72)	98 (97–98)	95 (93–96)	84 (83–85)	87 (86–88)

Abbreviations: CI, confidence interval; ESC, European Society of Cardiology; NPV, negative predictive value; PPV, positive predictive value.

**Table 3.** Sensitivity of 2015 and 2023 Duke–European Society of Cardiology Clinical Criteria in 476 Episodes of Confirmed Infective Endocarditis

Type of IE	Episodes, No.	Sensitivity (95% CI)	
		Duke-ESC 2015 Criteria	Duke-ESC 2023 Criteria
Confirmed	476	67 (63–72)	75 (71–79)
Prosthetic valve	120	70 (61–78)	73 (64–80)
Native valve	335	72 (66–76)	81 (77–85)

Abbreviation: CI, confidence interval, ESC, European Society of Cardiology; IE, infective endocarditis.

## DISCUSSION

The modifications implemented in the 2023 Duke-ESC criteria resulted in increased sensitivity compared with the 2015 version, whereas the specificity did not change [4]. However, with either version of the Duke-ESC clinical criteria, 33% of episodes remained classified as possible IE, thereby requiring clinicians to make the ultimate decision, thus representing a major limitation.

Three recent studies have examined the accuracy of the 2023 Duke-ESC criteria compared with the 2015 Duke-ESC criteria [5, 9, 12]. One study involved 595 Dutch patients with suspected IE, using the same cohort for validating the 2023 Duke-ISCVID criteria, the 2023 Duke-ESC, the 2015 Duke-ESC, and the modified Duke criteria [5]. Compared with the 2015 Duke-ESC criteria, the 2023 Duke-ESC criteria exhibited a higher sensitivity (80% vs 86%), but lower specificity (82% vs 94%). The same applied to the 2023 Duke-ISCVID criteria. The other study focused on a cohort of 1344 patients with *Staphylococcus aureus* IE [9]. The 2023 Duke-ESC criteria and the 2023 Duke-ISCVID showed improved sensitivity for *S. aureus* IE diagnosis (82% and 91%, respectively) compared with the 2015 Duke-ESC criteria (75%). However, the new Duke-ESC criteria exhibited reduced specificity (96% for both) compared with the 2015 criteria (99%). In the third study, focusing on episodes of streptococcal bacteremia, the 2023 Duke-ISCVID clinical criteria showed higher sensitivity for diagnosing IE compared with both Duke-ESC versions [12]. This increased sensitivity could be mainly attributed to the inclusion of *Streptococcus agalactiae* and *Streptococcus dysgalactiae* in the list of typical IE microorganisms by the ISCVID version.

The inclusion of leaflet thickening as an imaging criterion and of CIED and TAVI as cardiac predisposing factors improved the sensitivity of the 2023 Duke-ESC criteria. Leaflet thickening was observed in 9% of patients with diagnosed IE, in contrast to only 2% among those without IE (*P* < .001). In a previous study involving 86 cases of aortic bioprosthetic valve IE, leaflet thickening was detected in 13% of cases [13]. In the current study, among the 158 patients with IE and leaflet thickening, the majority (53%) also showed other typical IE lesions like vegetation or abscess. When excluding episodes with additional typical IE lesions, the incidence of leaflet thickening was found to be similar between IE and non-IE cases. Leaflet thickening can be present in various conditions, such as ageing-related degeneration, rheumatic or inflammatory valvular diseases, amyloidosis, and myxoid degeneration or prosthetic valve thrombosis, and it therefore lacks specificity [14, 15]. In a previous study among 78 healthy volunteers, TEE displayed leaflet thickening in 17 (22%) patients [16]. In addition, the machine settings may influence the appreciation of leaflet thickness on echocardiography (use of second harmonic imaging, inappropriate gain settings, etc). As a result, less experienced physicians may interpret the presence or absence of leaflet thickening ambiguously, which could affect the accuracy of the new criteria.

The only change to the microbiological criteria in the 2023 Duke-ESC criteria was the replacement of “community-acquired enterococci without known primary focus” with “*E. faecalis*” (*Enterococcus faecalis*). However, this modification proved to be disadvantageous for the new criteria. Compared with the 2015 Duke-ESC version, fewer patients with IE fulfilled the major microbiological criterion with the 2023 version (763 [65%] for 2023 vs 773 [66%] for 2015 criteria). Conversely, using the 2023 Duke-ESC criteria, more patients without IE fulfilled the major microbiological criterion compared with the 2015 version (675 [35%] for 2023 vs 648 [33%] for 2015 criteria). The revision to the 2023 Duke-ESC version was based on data from a multicenter Danish study, which revealed an IE prevalence of 26% among patients with *E. faecalis* bacteremia [17]. This rate was more than double that observed in all previous studies (ranging from 5% to 13%) [6, 18–20], surpassing the prevalence of IE among patients with *S. aureus* bacteremia [21–25].

The inclusion of hematogenous osteoarticular complications, such as spondylodiscitis or septic arthritis, is questionable, as none of these conditions has shown an association with IE. The lack of correlation with other embolic events raises doubts about categorizing them as vascular phenomena, echoing the rationale behind excluding cerebral microbleeds from the 2023 Duke-ESC criteria [11]. Moreover, osteoarticular complications predominantly result from hematogenous seeding during bacteremia rather than septic embolization of vegetations. In a previous study [26], IE was responsible for spondylodiscitis in only 12% of patients. Furthermore, the risk of IE depends on the timing of the onset of osteoarticular symptoms relative to the initiation of systemic symptoms. In another study of patients with *S. aureus* bacteremia linked to bone and joint infections, IE occurred in 23% of cases when osteoarticular symptoms preceded the onset of systemic symptoms by ≥4 days; conversely, if osteoarticular symptoms appeared ≥2 days after the onset of systemic symptoms, the rate of IE increased to 53% [27]. In a study including 1807 episodes of bacteremia by *S. aureus*, streptococci, and *E. faecalis*, the inclusion of spondylodiscitis under the vascular criterion led to the reclassification of 11 episodes from possible to definite IE, according to the 2023 Duke-ESC criteria. However, only 2 of these reclassified episodes were IE [28].

Recognizing its limitations, our study incorporated most of the patients retrospectively. Nevertheless, a substantial number of patients had suspicion of IE that was ultimately rejected, distinguishing the current study from previous research that primarily included confirmed IE cases [1, 3, 29]. Moreover, using both the endocarditis team (in prospective cohorts) and expert clinicians (in retrospective cohorts) could potentially result in misclassifications of episodes. The rationale for this approach to assessing IE cases stems from the lack of a definitive reference standard for IE diagnosis. This is primarily because IE demands a comprehensive and specialized approach to ensure precise diagnosis and successful management. In addition, the definition used for suspicion of IE was broad, encompassing patients with very low risk of IE. However, this mirrors actual clinical practice.

Our study was conducted in 2 Swiss hospitals, where infectious diseases specialists evaluate all suspected IE cases, with ready availability of ^18^F-FDG PET/CT and cardiac CT to evaluate valvular and paravalvular lesions, and cerebral and thoracoabdominal imaging studies to evaluate embolic events; our findings may therefore have limited generalizability to other healthcare systems. Furthermore, 724 patients (34%) underwent TTE exclusively. While TEE is superior to TTE in detecting IE, in patients with a low risk of IE and high-quality negative TTE findings, it may suffice to exclude IE [11, 30]. This scenario is relevant to nosocomial cases of *S. aureus* bacteremia lacking known risk factors for IE (such as history of prior IE, presence of CIED or prosthetic valves, persistent bacteremia for ≥72 hours, or embolic events), where the risk of IE was low (1%) [25].

In conclusion, the 2023 Duke-ESC clinical criteria revealed significant improvements in sensitivity compared with the 2015 version. However, the 2023 Duke-ESC version shares similar limitations to its predecessors, with approximately one-third of the episodes classified as possible IE. The additional elements introduced by the 2023 Duke-ESC criteria, such as leaflet thickening, osteoarticular septic manifestations, and *E. faecalis* as a typical pathogen, warrant further evaluation. In our study, their inclusion did not improve the discriminatory efficacy of the criteria; in fact, it may have even diminished it. The coexistence of 2 distinct iterations of the Duke criteria introduces complexity and potential confusion for clinicians. The necessity for judicious criterion selection in the presence of these dual versions could compromise diagnostic consistency and research coherence in the future.

## Notes

***Author contributions.*** M. P. O. and B. H. conceived the idea, supervised the project, and performed the analysis. M. P. O. wrote the first draft of the manuscript. All authors collected the patient data, interpreted the results, contributed to manuscript revision, and read and approved the submitted version.

***Financial support.*** This work was supported by the Swiss National Science Foundation (grants 32003B_219351/1 to M. P. O. and B. H. and 320030_184918/1 to B. H.) and the Clinical Research Priority Program (CRPP) of the University of Zurich (CRPP Precision medicine for bacterial infections; to B. H.).
